# The β-Secretase Substrate Seizure 6–Like Protein (SEZ6L) Controls Motor Functions in Mice

**DOI:** 10.1007/s12035-021-02660-y

**Published:** 2021-12-27

**Authors:** Emma Ong-Pålsson, Jasenka Rudan Njavro, Yvette Wilson, Martina Pigoni, Andree Schmidt, Stephan A. Müller, Michael Meyer, Jana Hartmann, Marc Aurel Busche, Jenny M. Gunnersen, Kathryn M. Munro, Stefan F. Lichtenthaler

**Affiliations:** 1 Department of Anatomy and Physiology, The University of Melbourne, Parkville, Victoria 3010 Australia; 2 German Center for Neurodegenerative Diseases (DZNE), Munich, Germany; 3 Neuroproteomics, School of Medicine, Klinikum rechts der Isar, Technical University of Munich, 81675 Munich, Germany; 4 Graduate School of Systemic Neurosciences, Ludwig Maximilian University, Munich, Germany; 5 Biomedical Center, Ludwig Maximilian University Munich, 82152 Planegg/Munich, Germany; 6 UK Dementia Research Institute at UCL, University College London, Great Britain, London, WC1E 6BT UK; 7 Institute of Neuroscience, Technical University of Munich, 80802 Munich, Germany; 8 The Florey Institute of Neuroscience and Mental Health, The University of Melbourne, Parkville, Victoria 3010 Australia; 9 Munich Cluster for Systems Neurology (SyNergy), Munich, Germany

**Keywords:** Seizure protein 6, DigiGait, Rotarod, Anxiety, Spatial learning and memory

## Abstract

The membrane protein seizure 6–like (SEZ6L) is a neuronal substrate of the Alzheimer’s disease protease BACE1, and little is known about its physiological function in the nervous system. Here, we show that SEZ6L constitutive knockout mice display motor phenotypes in adulthood, including changes in gait and decreased motor coordination. Additionally, SEZ6L knockout mice displayed increased anxiety-like behaviour, although spatial learning and memory in the Morris water maze were normal. Analysis of the gross anatomy and proteome of the adult SEZ6L knockout cerebellum did not reveal any major differences compared to wild type, indicating that lack of SEZ6L in other regions of the nervous system may contribute to the phenotypes observed. In summary, our study establishes physiological functions for SEZ6L in regulating motor coordination and curbing anxiety-related behaviour, indicating that aberrant SEZ6L function in the human nervous system may contribute to movement disorders and neuropsychiatric diseases.

**Supplementary Information:**

The online version contains supplementary material available at 10.1007/s12035-021-02660-y.

## Introduction

The protease β-site APP cleaving enzyme 1 (BACE1; also known as β-secretase) has fundamental functions in the nervous system, both under physiological and pathophysiological conditions. BACE1 is highly expressed in neurons and contributes to various physiological processes in the nervous system, including myelination, axon targeting and homeostasis of synapses [1, 2]. BACE1 is also linked to pathophysiological processes, in particular to Alzheimer’s disease where it is a major drug target because it cleaves the amyloid precursor protein (APP) and catalyzes the first step in the generation of the amyloid β peptide, a key pathogenic agent in Alzheimer’s disease [3]. BACE1-targeted inhibitors have advanced to phase 3 trials for Alzheimer’s disease. However, several of them, unexpectedly, induced side effects, including mild cognitive decline and psychiatric symptoms as well as an increased number of falls [4–7], pointing to potential defects in motor coordination. The molecular basis of the side effects is largely unknown, but they may result from too strongly inhibiting the cleavage of one or more of the numerous BACE1 substrates [8]. This issue needs to be resolved before clinical trials with BACE inhibitors are resumed. To date, the functions and consequences of BACE1 cleavage have only been studied for selected BACE1 substrates, such as type III neuregulin-1, Ig-containing β1 neuregulin, seizure protein 6 (SEZ6), close homolog of L1 (CHL1) and Aη, an APP-derived peptide [9–16].

For many other substrates and substrate candidates of BACE1, relatively little is known about their physiological function and how it may be altered through BACE1 cleavage. One of them is SEZ6-like (SEZ6L, also known as brain-specific receptor-like protein B (BSRP-B)), which forms a protein family with SEZ6 and SEZ6L2 that are also cleaved by BACE1 [9, 10, 17]. SEZ6L is broadly expressed in the murine brain, including in the neocortex and hippocampus as well as in the cerebellum [10, 17, 18], where SEZ6L is expressed in Purkinje and granule cells and in interneurons in the molecular layer of the cerebellum [17]. Given the strong expression of SEZ6L in the cerebellum, which contributes to motor coordination, SEZ6L may be involved in motor control in mice. In fact, mice lacking all three SEZ6 family members (SEZ6 triple knockout (KO) or TKO mice) have motor coordination deficits on the rotarod and cognitive deficits [17, 18]. These phenotypes appear less pronounced or absent for the single knockout mice of the SEZ6 family [17], suggesting that the three SEZ6 family members may have partially redundant functions although detailed analyses of an independently generated SEZ6 single KO mouse line revealed specific defects in motor coordination and cognition [19]. SEZ6L single KO mice have not yet been investigated in depth.

Here, we provide a detailed behavioural analysis of SEZ6L KO mice. We report that SEZ6L deficiency does not lead to major changes in the anatomy or proteome of the cerebellum. The lack of SEZ6L does induce specific deficits in motor functions as well as altered stress-responsive behaviour, although memory functions are not affected.

## Methods and Materials

### Animal Procedures

Mice were group-housed in standard conditions in the animal facility of the University of Melbourne. All experimental procedures accorded with the Australian Code of Practice for the Care and Use of Animals for Scientific Purposes and were approved by the Animal Ethics Committee of the University of Melbourne. Wild-type (WT), SEZ6L heterozygous (het) and SEZ6L knockout (KO) mice on a 129 × C57BL/6 background [17] were obtained from heterozygous matings. Additional WT and SEZ6L KO mice were housed in the pathogen-free animal facility of the Center for Stroke and Dementia Research (CSD) in Munich, Germany. Mouse work in the CSD was performed according to the European Communities Council Directive (86/609/EEC) and was approved by the committee responsible for animal ethics of the government of Upper Bavaria (02-19-067).

### Immunohistochemistry of the Cerebellum

We analysed 18-week-old SEZ6L KO and WT littermates (*n*=3). Animals were anesthetized intraperitoneally with a mixture of ketamine (400 mg/kg) and xylazine (27 mg/kg) and transcardially perfused with cold 0.1 M PBS for 5 min followed by 4% paraformaldehyde (PFA) in 0.1 M PBS for 15 min. Brains were isolated and post-fixed for 24 h in 4% PFA in 0.1 M PBS and afterwards kept until cutting in 0.5% PFA in 0.1 M PBS. Half of a cerebellum was glued to the stage of a Microm HM 650V vibratome (Thermo Scientific) and cut submersed in Ringer buffer in 25-μm parasagittal sections. Sections were stored at 4°C in 0.1 M PBS until staining. Free-floating sections were permeabilized, blocked and stained in PBS containing 0.4% Triton X100 and 5% BSA (PBS-T) together with the primary antibody overnight at 4°C. Sections were then washed 3 times with PBS-T and incubated with the appropriate secondary antibody (1:2000, goat anti-mouse Alexa 488 or donkey anti-rabbit Alexa 488, Invitrogen) together with nuclear stain DAPI (0.2 μg/ml, Roth) for 2 h at room temperature (RT). Sections were washed as before, mounted in Mowiol (Fluka) onto glass slides (Engelbrecht, Edermünde, Germany). Stained sections were stored at 4°C until analysis. For each primary antibody, sections from all animals were stained and processed in parallel using the same reagents. Genotypes were hidden during staining and analysis.

**Fig. 1:**
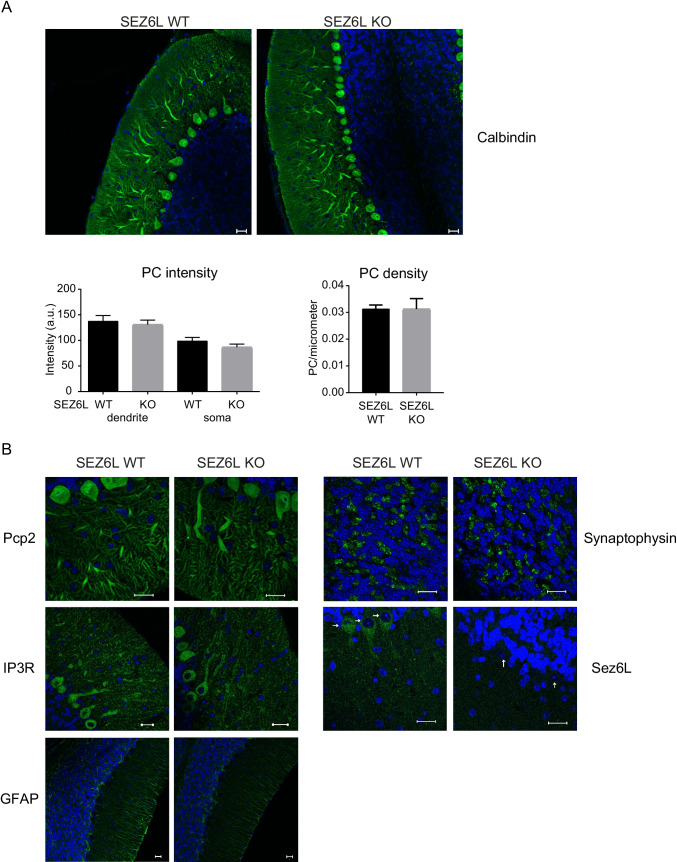
Immunohistochemistry of SEZ6L KO cerebellum. (A) Representative image of SEZ6L WT and KO Purkinje cells (PC) in cerebellar brain sections stained for calbindin. Staining intensity in PC dendrites and soma as well as PC density is not significantly different between the genotype in 3 biological replicates. Data displayed as mean ± SEM. a.u.: arbitrary units. (B) SEZ6L KO cerebellar sections did not show any difference in synaptic marker staining (synaptophysin), PC morphology (as stained with Pcp2), structure of the endoplasmic reticulum (as indicated by IP3R staining) nor in the glial marker GFAP. SEZ6L antibody was used as a control. White arrows represent Purkinje cells. Scale bar: 20 μm

Calbindin fluorescence intensity in Purkinje cell somata and dendrites was determined on calbindin (Cb38)-stained sections from z-stacks recorded using a ZEISS LSM710 confocal microscope equipped with Argon multiline and 405-nm diode lasers and a ×20, 0.8 (Fig. 1A, B: GFAP) or x63, 1.4 (Fig 1B: except GFAP) objective. Imaging parameters (except z-stack boundaries) were kept constant for a series of sections from all 6 animals stained with a given primary antibody. Comparisons between genotypes were performed within these series.

Measurements of mean intensity were calculated from the maximum intensity z-plane of circular ROIs within the apical part of five randomly selected Purkinje cell somata excluding nucleus and apical dendrites. For dendrites, measurements of mean intensity were calculated from the maximum intensity z-plane of rectangular ROIs within five randomly selected thick primary Purkinje cell dendrites. Quantification of Purkinje cell density was done on the upper or lower half of z-stacks selected for maximum intensity projection using the profile tool to measure the length of the PC layer. PC somata were counted manually.

Primary antibodies used: polyclonal rabbit anti-calbindin (Cb38, Swant), monoclonal SEZ6L (1:5; clone 21D9, IgG2a) [10], synaptophysin (1:100, mouse monoclonal, ab8049, Abcam), syntaxin 1B (1:500, rabbit polyclonal, Synaptic Systems), Pcp2 (1:4000, rabbit polyclonal, kind gift of Brad Denker), GFAP (1/4000, rabbit polyclonal, DAKO), IP3R (1:500, rabbit polyclonal, Alomone labs).

### Proteomic Analysis

Cerebella from 5-month-old WT and SEZ6L KO (*n*=4) were collected and lysed in STET buffer (50mM Tris pH 7.5, 150mM NaCl, 2mM EDTA, 1% TritonX-100, supplemented with 1:500 Protease inhibitor cocktail (Sigma)) as described [20–22] using the Precellys soft tissue lysis kit (Precellys). The mix of sample and ceramic beads was homogenized in the Precellys Evolution homogenizer with the following settings: 6500 rpm, cycle: 2 × 30s, pause: 3s. After a subsequent 15 min incubation step at 4 °C, samples were centrifuged at 16,000*g* and 4 °C for 15 min and the supernatant was transferred to a fresh tube.

Post-natal day 21 (P21) cerebella of the WT and SEZ6L KO (*n*=7) mice were collected and lysed in 1:1 ratio of high salt buffer (2M NaCl, 10 mM PBS pH 7.4, 1 mM EDTA) and STET buffer (50mM Tris pH 7.5, 150mM NaCl, 2mM EDTA, 2% TritonX-100) supplemented with 1:500 protease inhibitor cocktail (Sigma). Samples were processed using a tissue homogenizer (Omni International) at maximum speed for 60 s. Samples were incubated for 1h at 4 °C with occasional vortexing and afterwards centrifuged at 17,000*g* and 4 °C for 15 min. Supernatants were transferred to a fresh tube.

An amount of 25 units of Benzonase (Sigma-Aldrich) was added to 20 μg of protein and samples were incubated for 30 min at 37 °C at 1400 rpm in the Thermomixer (Eppendorf) to remove remaining DNA. Afterwards, samples were digested with LysC and trypsin, using single-pot, solid-phase-enhanced sample preparation (SP3) [23]. Proteolytic peptides were dried by vacuum centrifugation and dissolved in 20 μl 0.1% (v/v) formic acid.

Cerebella samples from 21 days were analysed using data-dependent acquisition. In total, 1.2 μg of peptides was separated on a nanoLC system (EASY-nLC 1200, Thermo Fisher Scientific) using an in-house packed C18 column (30 cm × 75 μm ID, ReproSil-Pur 120 C18-AQ, 1.9 μm, Dr. Maisch GmbH) with a binary gradient of water and 80% acetonitrile (B) containing 0.1% formic acid (0 min, 3% B; 3.5 min, 6% B; 137.5 min, 30% B; 168.5 min, 44% B; 182.5 min,75% B; 185 min, 99% B, 200 min, 99% B) at 50 C column temperature. The nanoLC was coupled online via a nanospray flex ion source equipped with a column oven (Sonation) to a Q-Exactive HF mass spectrometer (Thermo Fisher Scientific). Full MS spectra were acquired at a resolution of 120,000 and a *m/z* range from 300 to 1400. The top 15 peptide ions were chosen for collision-induced dissociation (resolution: 15,000, isolation width 1.6 *m*/*z*, AGC target: 1E+5, NCE: 26%). A dynamic exclusion of 120 s was used for peptide fragmentation).

Samples from 5-month-old mice were analysed using data-independent acquisition. Full MS spectra were acquired at a resolution of 120,000 (AGC target 5E+6). DIA fragmentation spectra were acquired by higher-energy collisional dissociation of all ions in 20 windows of variable size (resolution: 30,000, AGC target: 3E+6, stepped NCE 23.4%, 26%, 28.6%).

For spectral library generation, DDA measurements of mouse brain lysates (cerebellum and cerebrum) were performed. The raw data of DDA measurements were analysed with the Maxquant software (maxquant.org, Mack-Planck Institute Munich) [24] version 1.5.5.1.1.6.6.0. and searched against reviewed canonical FASTA database of Mus musculus (UniProt, 2018-07-23, 16,989 entries). Two missed trypsin cleavages were allowed. Oxidation of methionine and N-terminal acetylation were set as variable, carbamidomethylation of cysteine as static modifications. For the main search peptide and peptide fragment, mass tolerances were set to 4.5 and 20 ppm, respectively. Label-free protein quantification was performed on the basis of at least 2 ratio counts of unique peptides per protein.

The software Spectronaut (12.0.20491.14.21367) [25] was used with a self-generated library on murine brain tissues (82 runs, 201,130 precursors, 7988 protein groups), using the default settings. Briefly, a 1% FDR was applied to peptide and protein identifications and LFQ was performed on the MS2 level. LFQ of proteins required at least one identified peptide and quantification was performed with up to three peptides.

Generated LFQ outputs were log2-transformed and an average log2-fold change was calculated for each protein, which was identified in at least 3 biological replicates per experimental group. Changes in protein abundance were evaluated using Student’s *t*-test between the log2 LFQ intensities of the two experimental groups. A permutation-based FDR estimation was used to account for multiple hypotheses (*p*=5%; s0=0.1) using the software Perseus [26, 27].

### Behavioural Testing

Male and female WT, SEZ6L het and SEZ6L KO mice aged between 4 and 6 months were used. Littermates were used and groups were age-matched. Experiments were performed in the light cycle and mice were acclimatised to the testing room, and equipment as appropriate, before behavioural testing. One cohort underwent multiple tests in a 2-month period, between 4 and 6 months of age, and tests were performed in the following order: inverted screen test, forelimb grip strength, ledged beam, DigiGait, locomotor cells, light/dark box and Morris water maze (only WT and SEZ6L KO underwent this final test). Mice tested in cohort one (excluding Morris water maze): 23–24 WT (9 male, 14–15 female), 30 SEZ6L het (15 male, 15 female) and 23 SEZ6L KO (15 male, 8 female). A number of additional mice were tested on the ledged beam and DigiGait; numbers are indicated in the relevant figure legend. Mice tested in Morris water maze: 22 WTs (9 male, 13 female) and 21 SEZ6L KOs (13 male, 8 female). A second cohort of mice aged 4–5 months was tested on the rotarod only: 29 WT (15 male, 14 female), 24 SEZ6L het (18 male, 16 female) and 36 SEZ6L KO (21 male, 15 female). A third cohort of mice aged 3–5 months was tested on the elevated open field: 30 WT (17 male, 13 female), 33 SEZ6L het (17 male, 16 female) and 29 SEZ6L KOs (15 male, 14 female). All training and testing sessions were performed by investigators blinded to the genotype of the mice. Statistical analysis was performed using GraphPad Prism (GraphPad Software, Inv.) as described below and in figure legends. Values represent mean ± standard error of mean (SEM). Data from male and female mice were pooled in analyses where there was no statistical evidence of a sex difference.

#### Gait Analysis

Gait analysis was quantified with DigiGait imaging apparatus and software (Mouse Specifics Inc., Boston, MA). Mice were placed on a transparent treadmill within a 15 × 5 cm plexiglass compartment. Prior to the main study, a separate cohort of mice from the same colony was tested on a range of treadmill speeds and 25 cm/s was identified as a fast speed which allowed most mice to maintain a gait free from contact with the rear bumper or wall. A video camera mounted underneath the treadmill belt captured ventral images of the mice running at constant speeds of 15 cm/s and 25 cm/s and the videos (4 s long) were analysed with DigiGait software as previously described [18]. One male SEZ6L het was unable to run at a speed of either 15 or 25 cm/s for a sufficient length of time for analysis to be completed. Ten mice (3 male WT, 2 female WT, 1 male SEZ6L het, 2 male SEZ6L KOs and 2 female SEZ6L KOs) were unable to run for a sufficient length of time at 25 cm/s.

#### Rotarod

Motor coordination and learning were assessed by testing mice on an accelerating rotarod (IITC Life Science Inc.) with cylinders 1.25 inches in diameter. The rotarod speed gradually increased from 1 to 40 RPM over 5 min. Mice underwent 3 trials per day for 5 consecutive days with an intertrial interval of approximately 10 min. The latency to fall was recorded as the time the mouse fell off the cylinder or did one full passive rotation. If a mouse remained on the cylinder at the end of the trial, the latency was recorded as 300 s.

#### Assessment of Muscle Strength

Forelimb grip strength was recorded on a mouse grip strength meter (Ametek, USA). Mice were lifted by the tail, allowed to grasp the triangular pull bar with both forepaws and were gently pulled backwards in the horizontal plane until their forepaws released the bar. The peak tension (in kg) was recorded for five successful tests performed 30 s apart. The highest value from the five trials was recorded as the grip strength for that mouse and was normalised to its body weight. In the inverted screen test, mice were placed on a 20cm × 20 cm grid screen composed of 1cm × 1cm squares of 1mm diameter wire. The screen was held 40 cm above an enclosed area containing cushioning and after ~5 s was slowly inverted so the mice were hanging upside down with all four paws gripping the screen. The time until the mouse fell off the screen was recorded as the ‘hang time’. After a maximum of 600 s, any mice still gripping the screen were removed.

#### Ledged Beam

Locomotor precision was assessed with the ledge beam. Mice were placed onto an 80-cm-long black Perspex beam that was 3.5cm wide at the starting end and progressively narrowed to 1mm. One centimeter below the beam was a 0.5cm wide clear plastic ledge that the mice could use to recover if their feet slipped off the beam. Mice performed 2 days of training with 3 traversals per day prior to testing. On the test day, mice traversed the beam once while being recorded. Videos of the left and right sides were analysed for forepaw and hindpaw foot faults, total number of steps (hindpaw placements) and total time to traverse the beam (s).

#### Locomotor Cells

Mice were individually placed in activity test chambers (Med Associates Inc.) measuring 27.5 × 27.5 cm × 20.5 cm (height) for 30 min under ambient lighting. Locomotor activity was recorded for 30 min and analysed in the horizontal and vertical planes.

#### Light/Dark Box

Anxiety-like behaviour was investigated with the light/dark box test. Mice were individually placed in activity test chambers as described above with a black plastic insert that created a dark area in one half of the chamber (27.5 cm × 13.5 cm) with an opening for the mouse to move between light and dark areas. The ‘light’ part of the chamber was illuminated to 750 lux. Mice were placed in the dark area and the locomotor activity in the light and dark areas was recorded for 10 min.

#### Elevated Open Field

Anxiety-like behaviour in an aversive environment was assessed with the elevated open field as previously described [28]. Mice were placed on the test arena (75 × 100cm) without walls situated 60cm above the ground. Overhead lighting was switched off and two spotlights (3000 lux) on either side of the arena shone directly onto the field to create an aversive environment. Mice were placed in the centre of the field and allowed to explore freely for 3 min. Videos were obtained using TopScan Lite (CleverSys Inc.). Time moved and latency to leave the centre were recorded by the experimenter.

#### Morris Water Maze

Spatial learning and memory were assessed with the Morris water maze. A pool measuring 1.4 m in diameter was surrounded by spatial cues. The water depth was 30 cm with non-toxic white paint added to make the water opaque. The ‘hidden’ platform (15 cm diameter) was submerged 1 cm below the water level. In the acquisition phase, mice were placed in a given quadrant and given up to 2 min to find the platform; after this time, the mouse was gently guided to the platform. Mice spent 10 s on the platform before they were removed, had excess water gently blotted off and were placed in a cage under a warming lamp to dry. Mice had four acquisition sessions within a 1-h period at approximately the same time each day for 6 days. During the acquisition phase, the starting coordinate (north/east/west/south) changed with each session on a given day and the order of starting coordinates changed daily; the location of the hidden platform (NE quadrant) remained the same. In the probe trial on day 7, mice were placed in the SW quadrant and the platform was removed; mice were left in the pool for 1 min and the amount of time spent in/distance travelled in/entries into the target NE quadrant was observed. Cognitive flexibility was examined by altering the platform location. The reversal phase was conducted in the same way as the acquisition phase with the location of the hidden platform moved (to the SW quadrant). Mice had four reversal sessions within a 1-h period at the same time each day for 4 days. In the reversal probe trial on day 12, mice were placed in the NE quadrant and the platform was removed; mice were left in the pool for 1 min and the amount of time spent in/distance travelled in/entries into the target SW quadrant was observed. Behavioural parameters were analysed with CleverSys Topscan tracking software.

## Results

### SEZ6L Deficiency Does Not Alter Cerebellar Anatomy and Proteome

SEZ6L-deficient (SEZ6L KO) mice were generated as described [17]. SEZ6L het and KO mice were viable and had no obvious health issues. Within sex, there was no significant effect of genotype on body weight in 4-month-old male mice (WT 30.2 ± 1.3 g, SEZ6L het 30.3 ± 0.7 g, SEZ6L KO 31.3 ± 0.9 g; 1-way ANOVA *p*>0.05, *n*=13–16/genotype); however, 4-month-old female SEZ6L het and SEZ6L KO mice were slightly heavier than female WTs (WT 21.2 ± 0.5 g, SEZ6L het 24.4 ± 0.7 g, SEZ6L KO 23.4 ± 0.7 g; 1-way ANOVA *p*=0.0030, *n*=15–16/genotype).

Because SEZ6L is highly expressed in the cerebellum [17, 18], we used immunohistochemistry and proteomics to determine whether SEZ6L deficiency induces major changes in cerebellar anatomy or the cerebellar proteome. Sections from wild-type and SEZ6L KO mice at 4 months of age were stained for (a) calbindin, (b) Purkinje cell–specific protein 2 (pcp2), which serve as markers of the cerebellar Purkinje cells (PC), (c) for inositol 1,4,5-trisphosphate (IP3) receptor, which is highly expressed in PCs, (d) for glial fibrillary acidic protein (GFAP), which is a marker for astrocytes and (e) for the neuronal protein synaptophysin (Fig. 1A, B). As a control, SEZ6L staining was seen in wild-type, but not in SEZ6L KO cerebellum (Fig. 1B). The intensity of the calbindin staining was not different between the genotypes, in either the PC dendrites or in the soma (Fig. 1A). Likewise, the density of PCs within the cerebellum was not changed (Fig. 1A). Similarly, staining for Pcp2, IP3, GFAP and synaptophysin did not reveal obvious differences between wild-type and SEZ6L KO mice. Thus, we conclude that SEZ6L deficiency does not induce major neuroanatomical changes in the cerebellum.

**Fig. 2:**
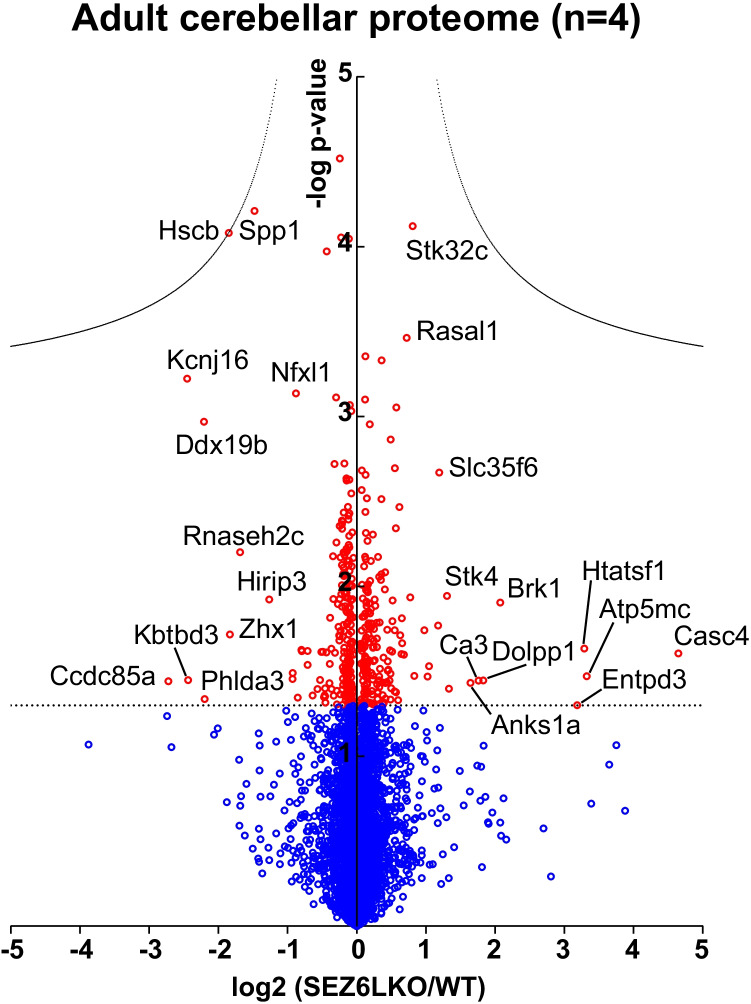
SEZ6L KO cerebellar proteome. Volcano plot of SEZ6L KO and WT cerebella with four biological replicates. The mean protein log2-transformed fold changes between SEZ6L KO and WT cerebella of each protein are plotted against the negative log10-transformed *p*-value. Proteins with a *t*-test *p*-value < 0.05 are shown as red circles. Proteins that remain significant after permutation-based FDR correction (FDR <0.05, s0= 0.1) are located above the hyperbolic curves. The straight dotted line crosses the y-axis at 1.3 and represents the *p*-value of 0.05.

Next, we used quantitative, label-free mass spectrometry–based proteomics to compare the proteome of the cerebellum from 5-month-old wild-type and SEZ6L KO mice (Supp. Table 1). Total cerebellar protein extracts of four biological replicates of each genotype were analysed. Protein abundance differences between SEZ6L KO mice and wild-type mice are displayed in a volcano plot (Fig. 2). Proteins with reduced abundance in the SEZ6L KO cerebellum are on the left side of the y-axis, whereas proteins with an increased abundance are on the right side of the y-axis. Proteins indicated with a circle in red have a *p*-value of less than 0.05. The hyperbolic curves correct for multiple hypothesis testing using a false discovery rate (FDR) approach. While numerous proteins appeared to have reduced or increased levels in the SEZ6L KO cerebellum, none of the proteins remained statistically significant after FDR correction (Fig. 2). Similar results were obtained from young mice at post-natal day 21 (P21) (Suppl. Fig. 1). We thus conclude that loss of SEZ6L does not induce major changes of the murine cerebellar proteome.

### SEZ6L KO Mice Exhibit Gait Differences as Assessed by Treadmill Walking

SEZ6 TKO mice lacking SEZ6, SEZ6L and SEZ6L2 have a distinct motor phenotype [17, 18]. Although we found that SEZ6L deficiency did not lead to major anatomical or proteomic changes in the cerebellum, it is important to note that the cerebellum is only one of several anatomical structures involved in motor control. Thus, a detailed behavioural analysis of SEZ6L KO mice is required to test for the involvement of SEZ6L in motor control. To assess the role of SEZ6L in normal movement parameters, we employed the DigiGait system which is widely used to analyse gait in genetically altered mice. Experimental mice were first tested at a treadmill speed of 15 cm/s and then at 25 cm/s, and, for most DigiGait indices, average forelimb/paw and average hindlimb/paw values for each mouse were used for analysis. Animal body width did not vary significantly between genotypes or sexes (average of DigiGait measurements taken at 15 and 25 cm/s; 2-way ANOVA with genotype and sex as factors). There was a significant effect of sex, but not genotype, on animal length from nose to base of the tail (2-way ANOVA, *p* = 0.0044) with males slightly longer than females (13.3 ± 0.136 vs. 12.7 ± 0.168 cm). In order to identify gait parameters that exhibit sex-specific changes, major DigiGait indices were analysed with a 2-way ANOVA with genotype and sex as factors (Supp. Table 2). Where no sex difference was observed, data from male and female mice were pooled.

SEZ6L KO mice exhibited a number of gait differences to WT mice, and in some cases to SEZ6L het mice, at treadmill speeds of 25 cm/s (Table 1) and 15 cm/s (Supp. Table 3). On a treadmill speed of 25 cm/s, SEZ6L KO mice exhibited increased stride length (~13% higher in KO compared to WT), increased time to complete a stride (~11% higher) and consequently a decrease in stride frequency (steps per second; ~11% lower). While data from male and female mice were pooled for this analysis, this change in gait was comparable in male and female SEZ6L KO mice when sexes were analysed separately (e.g. hindlimb stride length in male WT vs. KO = 6.22 ± 0.246 vs. 6.78 ± 0.175; hindlimb stride length in female WT vs. KO = 5.73 ± 0.264 vs. 6.60 ± 0.292). At the slower speed of 15 cm/s, changes to stride time and frequency were similarly altered in SEZ6L KO compared to WT mice and stride length was significantly increased in SEZ6L KO compared to het mice when examining hindlimbs. Stride time is a combination of stance duration (paw contact with treadmill) and swing time (no paw contact); both of these indices were increased in some SEZ6L KO categories at both treadmill speeds. Stance time can be further divided into brake duration (initial to maximal paw contact) and the propulsion phase. These indices were not significantly different between genotypes at 25 cm/s apart from an increase in propulsion time of the hindlimbs between male SEZ6L KO and het mice. At 15 cm/s, brake duration was increased in SEZ6L KOs compared to WT (forelimb) and in SEZ6L hets compared to WT (hindlimb). There was no difference in stance width between genotypes, indicating no deficit in stability in SEZ6L KOs. Overall, the gait of SEZ6L KO mice primarily differs from WTs in stride length and frequency: SEZ6L KO mice take longer and less frequent strides than WT mice to maintain the treadmill pace.

**Table 1:** SEZ6L KO mice exhibit gait differences as assessed by treadmill walking at 25 cm/s. *p*-values generated from 1-way ANOVA with male and female data pooled unless otherwise indicated. Tukey’s multiple comparisons test significant differences between WT vs. SEZ6L KO are indicated with an asterisk (*) and SEZ6L het vs. SEZ6L KO indicated with a hash (#). *p ≤ 0.05; **p ≤ 0.01; ***p ≤ 0.001 (or equivalent symbol). Data presented as mean ± SEM. WT *n* = 19 (6 male, 13 female), SEZ6L het *n* = 28 (13 male, 15 female) and SEZ6L KO *n* = 20 (13 male, 7 female). *n.s.* = not significant (*p*>0.05)

DigiGait indices	Examination of forelimb/paw or hindlimb/paw	*p*-value	WT	SEZ6L het	SEZ6L KO
Stance width (cm)	Between forelimbs	n.s.	1.679 ± 0.042	1.675 ± 0.040	1.730 ± 0.048
	Between hindlimbs	n.s.	2.921 ± 0.066	2.979 ± 0.043	2.860 ± 0.058
Stride length (cm)	Forelimb average	0.0013	5.87 ± 0.196	6.26 ± 0.083	6.65 ± 0.134 ***
	Hindlimb average	0.0006	5.88 ± 0.200	6.27 ± 0.075	6.72 ± 0.150 *** and #
Stride frequency (steps/s)	Forelimb average	0.0024	4.31 ± 0.101	4.10 ± 0.054	3.88 ± 0.091 **
	Hindlimb average	0.0006	4.35 ± 0.101	4.11 ± 0.049	3.86 ± 0.097 ***
Stride time (s)	Forelimb average	0.0017	0.240 ± 0.005	0.251 ± 0.003	0.266 ± 0.005 **
	Hindlimb average	0.0005	0.239 ± 0.006	0.251 ± 0.003	0.269 ± 0.006 *** and #
Stance duration (s)	Forelimb average	0.0005	0.147 ± 0.002	0.155 ± 0.002	0.165 ± 0.004 *** and #
	Hindlimb average—males	n.s.	0.161 ± 0.007	0.167 ± 0.003	0.175 ± 0.003
	Hindlimb average—females	n.s.	0.155 ± 0.003	0.162 ± 0.003	0.163 ± 0.006
Swing duration (s)	Forelimb average	n.s.	0.093 ± 0.004	0.095 ± 0.002	0.100 ± 0.003
	Hindlimb average	0.0044	0.083 ± 0.003	0.087 ± 0.002	0.098 ± 0.004 ** and #
Propulsion phase (s)	Forelimb average—males	n.s.	0.101 ± 0.006	0.102 ± 0.004	0.107 ± 0.004
	Forelimb average—females	n.s.	0.088 ± 0.003	0.096 ± 0.004	0.097 ± 0.004
	Hindlimb average—males	0.0445	0.127 ± 0.005	0.126 ± 0.005	0.140 ± 0.004 #
	Hindlimb average—females	n.s.	0.118 ± 0.004	0.118 ± 0.004	0.119 ± 0.005
Brake duration (s)	Forelimb average	n.s.	0.054 ± 0.002	0.056 ± 0.002	0.062 ± 0.003
	Hindlimb average	n.s.	0.036 ± 0.003	0.042 ± 0.003	0.038 ± 0.003

### SEZ6L KO Mice Show Motor Coordination Deficits on the Accelerating Rotarod

**Fig. 3:**
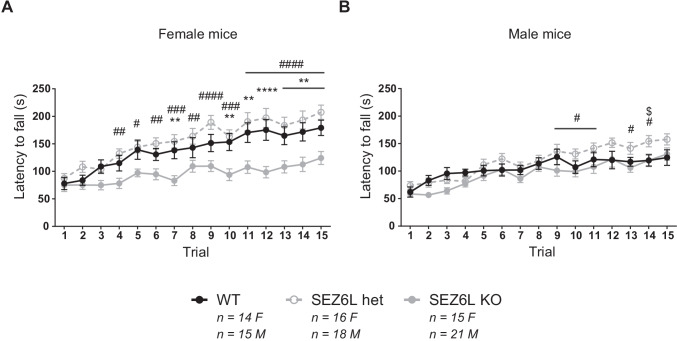
SEZ6L KO mice show motor coordination deficits on the accelerating rotarod. On the accelerating rotarod (1–40 rpm, 3 trials/day over 5 days), female SEZ6L KO mice performed significantly worse than both WT and SEZ6L het mice (A). In contrast, male SEZ6L KO and WT mice performed similarly, although differences were seen between SEZ6L het and KO mice in later trials and between SEZ6L het and WT mice in trial 14 (B). Female cohort: WT *n* = 14, SEZ6L het *n* = 16, SEZ6L KO *n* = 15. Male cohort: WT *n* = 15, SEZ6L het *n*=18, SEZ6L KO *n* = 21. Data analysed with repeated measures 2-way ANOVA and shown as mean ± SEM. Significant WT vs. SEZ6L KO differences indicted with an asterisk (*), SEZ6L het vs. SEZ6L KO differences indicated with a hash (#) and WT vs. SEZ6L het differences indicated with $. *p ≤ 0.05; **p ≤ 0.01; ***p ≤ 0.001; ****p ≤ 0.0001 (or equivalent symbol)

On the accelerating rotarod (1–40 RPM, 3 trials/day over 5 days), SEZ6L KO females did not exhibit the same degree of motor improvement as WT and SEZ6L het female mice over the course of the experiment, having a shorter latency to fall. In the female cohort, there were significant effects of genotype (*p* = 0.0001), trial (*p* < 0.0001) and interaction between factors (*p*=0.0001; 2-way repeated measured ANOVA). Tukey’s multiple comparisons test indicated differences between WT and SEZ6L KO females on trials 7 and trials 10–15. Similarly, SEZ6L KO females had a shorter latency to fall than SEZ6L het females during trials 4–15. No significant differences were seen between SEZ6L het and WT females (Fig. 3A). Compared to the striking deficit of female SEZ6L KO mice on the rotarod, differences between male genotypes were minimal. In the male cohort, there were no significant effects of genotype (*p* = 0.0722); however, there were significant effects of trial (*p* < 0.0001) and interaction between factors (*p* = 0.0156; 2-way repeated measured ANOVA). Tukey’s multiple comparisons test indicated no differences between WT and SEZ6L KO males in individual trials. There were differences between SEZ6L het and KO males from trials 9 to 11 and 13 to 14 and between SEZ6L het and WT males at trial 14; in these trials, SEZ6L het males remained on the rotarod for a longer period of time (Fig. 3B). In summary, SEZ6L KO mice displayed deficits in motor coordination and learning on the accelerating rotarod in a sex-specific manner.

### SEZ6L KO Mice Exhibit Normal Spontaneous Movement, Locomotor Precision and Muscular Strength

**Fig. 4:**
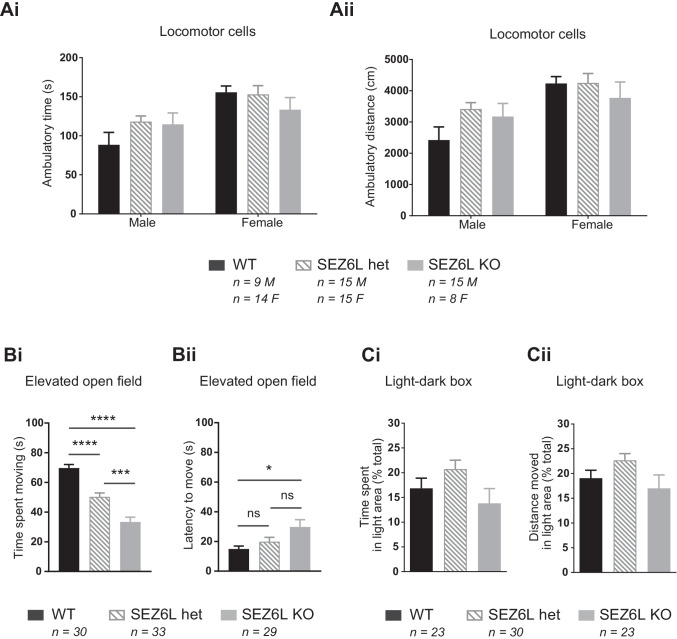
SEZ6L KO mice exhibit normal spontaneous locomotion and increased anxiety-like behaviour. WT, SEZ6L het and SEZ6L KO mice behave similarly in locomotor cells (Ai-ii: 1-way ANOVA within sex). Mice tested: 23 WTs (9 male, 14 female), 30 SEZ6L hets (15 male, 15 female) and 23 SEZ6L KOs (15 male, 8 female). In the elevated open field, the time spent moving was approximately halved in SEZ6L KO compared to WT mice. The time spent moving by SEZ6L hets was part way between the WT and SEZ6L KO groups (Bi). SEZ6L KO mice took longer to leave the centre of the field at the beginning of the test compared to WT mice (Bii). WT *n* = 30, SEZ6L het *n* = 33 and SEZ6L KO *n* = 29. Data analysed using one-way ANOVA (Bi) and Kruskal-Wallis test (Bii) and shown as mean ± SEM. *p ≤ 0.05; ***p ≤ 0.001; ****p ≤ 0.0001. A cohort of naive mice was tested in the elevated open field only, as outlined in the ‘Methods and Materials’ section. In the light-dark box, there was no significant difference between genotypes in the proportion of time spent (Ci) or distance moved (Cii) in the light area over 10 min, indicating no change in anxiety. Data analysed as 1-way ANOVA and displayed as mean ± SEM. WT *n* = 23, SEZ6L het *n* = 30, SEZ6L KO *n* = 23

SEZ6L KO, het and WT mice exhibited similar levels of spontaneous movement in locomotor cells, as assessed by their ambulatory time (Fig. 4A) and distance (Fig. 4B) over either 5 or 30 min (5-min data not shown). SEZ6L KO mice did not display deficits in locomotor precision on the ledged beam, an apparatus that becomes progressively narrower in length. No difference was observed between SEZ6L KO, het and WT mice in forepaw or hindpaw errors per step (Supp. Fig. 2Ai-ii) and all genotypes had a similar beam traversal time (not shown). Additionally, there was no difference between genotypes in the total number of steps taken to traverse the ledged beam (not shown). There was no difference between SEZ6L KO, het and WT mice in the peak strength (units) relative to body mass as determined by the forelimb grip strength test (Supp. Fig. 2B) and no difference between genotypes in hang time on the inverted screen test (Supp. Fig. 2C), indicating normal muscular strength.

### SEZ6L KO Mice Exhibit Enhanced Anxiety-Like Behaviour

SEZ6 TKO mice lacking SEZ6L and related family members showed increased stress responsiveness on the elevated open field and deficits in spatial memory [18]; therefore, SEZ6L KO mice underwent these behavioural analyses. A cohort of naïve mice was tested on the elevated open field, an aversive environment with no walls and bright lights which elicits anxiety-like behaviour in mice [18, 28]. A clear difference was seen in exploratory behaviour across genotypes; the time spent moving by SEZ6L KO mice in this 3-min test was approximately half the time spent moving by WTs, and SEZ6L het mice displayed a level of movement part way between the SEZ6L KO and WT groups (Fig. 4Bi; 1-way ANOVA. Tukey’s multiple comparisons test: KO vs. WT, *p* < 0.0001; KO vs. het, *p* = 0.001; het vs. WT, *p* < 0.0001. KO = 33.4 ± 3.2 s, het = 50.3 ± 2.6 s, WT = 69.7 ± 2.4 s). SEZ6L KO mice took significantly longer to leave the centre of the field at the beginning of the test compared to WT mice (Fig. 4Bii; non-parametric Kruskal-Wallis 1-way ANOVA. SEZ6L KO vs. WT, *p* = 0.0355, 29.8 ± 4.9 s vs. 14.9 ± 2.0 s). In contrast, similar levels of anxiety were displayed by WT, SEZ6L het and SEZ6L KO mice as measured by their behaviour in the less aversive light-dark box test (Figure 4C). There was no difference between genotypes in the time spent in the light area (as % duration of total time, Fig. 4Ci) at either 5 or 10 min (5-min data not shown) and no difference between genotypes in the distance moved in the light area (as % duration of total distance, Fig. 4Cii) at either 5 or 10 min (5-min data not shown). In contrast to their behaviour in the locomotor cells, there was a slight decrease in the overall level of movement exhibited by SEZ6L KO compared to SEZ6L het mice when looking at the total time spent moving and total distance moved in the whole apparatus (10-min timepoint; time (s): WT 52.3 ± 3.63, SEZ6L het 60.1 ± 2.59, SEZ6L KO 44.4 ± 4.39, *p* = 0.0073; distance (mm): WT 1617 ± 107, SEZ6L het 1834 ± 68.8, SEZ6L KO 1358 ± 140, *p* = 0.0062, 1-way ANOVA with sex pooled). In summary, the behaviour in the aversive environment of the elevated open field indicates altered stress responsiveness in both SEZ6L KO and het mice compared to WT.

### SEZ6L KO Mice Perform Normally in a Test of Spatial Learning and Memory

**Fig. 5:**
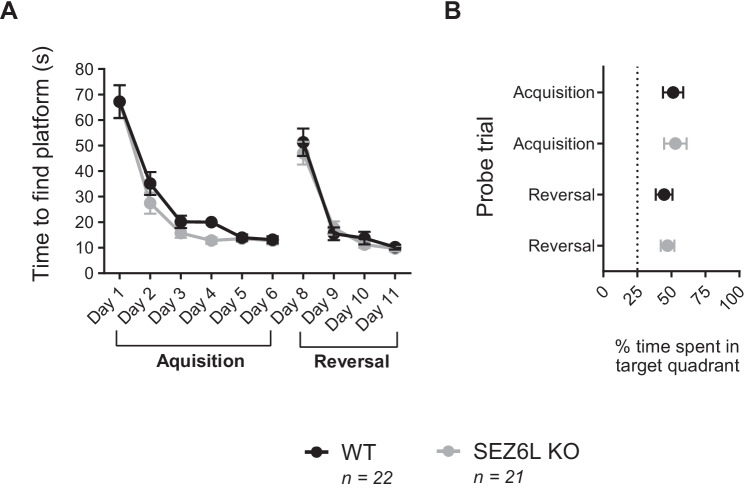
SEZ6L KO mice perform normally in a test of spatial learning and memory. WT and SEZ6L KO mice perform similarly in acquisition and reversal phases of the Morris water maze in both the time taken to learn the location of the hidden platform (A, data analysed with repeated measures 2-way ANOVA and displayed as mean ± SEM) and in the proportion of time spent in the target quadrant during probe trials (B, displayed as mean with 95% confidence interval; chance level indicated by dotted line). WT *n* = 22 (9 male, 13 female), SEZ6L KO *n* = 21 (13 male, 8 female)

In the Morris water maze, a test of spatial learning and cognitive flexibility, WT and SEZ6L KO mice performed similarly (het mice were not tested). In both the acquisition (days 1–6) and reversal (day 8–11) phases, there was a significant effect of day (*p* < 0.0001) but no effect of genotype or interaction of factors (*p* > 0.05, 2-way repeated measures ANOVA) when looking at the amount of time taken to find the hidden platform (Fig. 5A). There was also no effect of genotype on the path length distance taken to find the platform (data not shown) and no difference between genotypes in swimming velocity (mm/s, data not shown), indicating that the gait differences seen in SEZ6L KO mice do not affect their swimming speed. WT and SEZ6L KO mice both spent an increased percentage of time in the target quadrant in both the acquisition and reversal probe trials (as determined by 95% confidence interval of the mean time not overlapping with chance; Fig. 5B), demonstrating that both genotypes learnt the task initially and when the platform position was altered. These results suggest that constitutive SEZ6L deletion does not result in spatial learning and memory deficits.

### Comparison of SEZ6L KO and SEZ6 TKO Phenotypes

SEZ6 TKO mice lacking SEZ6, SEZ6L and SEZ6L2 display motor and cognitive deficits [18] and these are summarised in Table 2. Similar but less pronounced motor deficits have now been identified in SEZ6L KO mice, while results from the elevated open field are comparable in SEZ6L KO and SEZ6 TKO mice. SEZ6L KO mice did not display the reversal learning deficits in the Morris water maze seen in SEZ6 TKOs (Table 2).

**Table 2:** Comparison of motor and cognitive phenotypes seen in SEZ6L KO and SEZ6 TKO mice [18]

Behavioural test	Measurement	Result in SEZ6L KO compared to WT	Result in SEZ6 TKO compared to WT [18]
DigiGait—25cm/s speed	Stance width	No difference (male and female mice tested)	Wider stance width between forepaws and hindpaws (male and female mice tested)
	Stride length	Increased	Increased
	Stride frequency	Decreased	Decreased
	Stride time/stance duration/swing duration	Stride time increased; stance and swing components increased	Stance and swing components increased
	Propulsion/brake duration	No difference	Braking time reduced; propulsion increased
Accelerating rotarod *	Latency to fall	Females had decreased latency to fall; no difference in male cohort	Females not tested; males had decreased latency to fall
Ledge beam	Foot faults	No deficit in male or female mice	More hindpaw faults (male mice tested)
	Traversal speed	No difference	Slower traversal speed
Locomotor cells	Distance moved	No difference in male or female mice	Decreased by ~50% (male mice tested)
Grip strength	Forelimb force exertion	No deficit in male or female mice	Slight deficit (male mice tested)
Inverted screen test	Hang time	No deficit in male or female mice	Decreased latency to fall (male mice tested) #
Elevated open field	Time spent moving	Decreased (~50%) in male and female mice	Decreased (~50%) in male and female mice
	Latency to move from centre	Increased	Increased
Morris water Maze	Acquisition phase	No deficit (male and female mice)	No deficit in path length (male and female mice)
	Reversal phase	No deficit	Reversal learning deficit

*Rod diameter was ~3.2 cm in the current study and 9.5 cm in [18]. Rods accelerated to a maximum of 40 RPM in the current study and 23 RPM in [18]

#Nash, A. N (2019). Investigating the role of Seizure related gene 6 family proteins and their BACE shed products at excitatory synapses: impacts on motor and cognitive function. Unpublished PhD thesis. The University of Melbourne, Australia

## Discussion

Our study reports new functions for SEZ6L in the nervous system. We establish SEZ6L as a gene essential for normal motor coordination and important for controlling body movements. We further demonstrate that the lack of SEZ6L is associated with anxiety-related behaviour, indicating that SEZ6L loss of function may contribute to aspects of neuropsychiatric diseases.

Among members of the SEZ6 family, substantial knowledge has been gained about SEZ6, which has a fundamental role in the nervous system, e.g. in synaptic connectivity, motor coordination, synaptic transmission through kainate receptors and long-term potentiation, a cellular correlate of learning and memory [19, 29, 30]. Altered SEZ6 levels in cerebrospinal fluid are also linked to neuropsychiatric and neurodegenerative disorders, including Alzheimer’s disease [31, 32]. Compared to SEZ6, much less is known about SEZ6L. While the expression of SEZ6 is largely restricted to neurons [19], SEZ6L is expressed more widely, both within and outside of the brain, including in the pancreas [10, 17, 18, 33]. Variants of the SEZ6L gene are linked to distinct neuropsychiatric diseases, such as bipolar disorder and autism spectrum disorders [34, 35]*,* and also to cancer (e.g. [36]). To date, little is known about how changes in SEZ6L contribute to the pathogenesis of these diseases. At the molecular level, SEZ6L is a complement regulator, raising the possibility that SEZ6L is involved in complement-dependent synaptic pruning during central nervous system development [37].

Our study now establishes a major function for SEZ6L in controlling motor coordination in mice. The changes in gait observed in SEZ6L KOs, namely the longer and less frequent strides, were also observed in SEZ6 TKO mice, lacking all three SEZ6 family members [18], which have additional motor deficits (Table 2). This suggests that the gait abnormalities seen in SEZ6 TKO mice may be primarily due to the loss of SEZ6L, although whether a single knockout of SEZ6 or SEZ6L2 results in a similar gait phenotype has not been examined. Expression of SEZ6L is high in multiple areas of the developing and adult brain that are important for motor function and the mechanisms underlying the altered gait in SEZ6L KO mice are yet to be determined. While we observed no changes in the proteome of the cerebellum in SEZ6L KO mice, future proteomic studies should assess other motor areas expressing SEZ6L, such as the motor cortex, or examine cerebellar changes in a more sensitive way, for example by micro-dissecting the deep cerebellar nuclei. The gait differences observed between SEZ6L KO and WT mice were similar in males and females; likewise, the gait phenotype in SEZ6 TKO mice was not sex-specific [18]. In contrast, a sex-specific SEZ6L KO phenotype was seen on the rotarod: female SEZ6L KO mice had a significant deficit compared to female SEZ6L het/WT mice but males of all genotypes performed similarly. It should be noted that the improvement of male mice over the course of the experiment was relatively poor which raises the possibility that the rotarod parameters, while appropriate for the female cohort, may have been too challenging for the males and, therefore, less suitable for detecting differences between genotypes in the male cohort. SEZ6L het and KO female mice were slightly heavier than WTs; however, this did not negatively influence the performance of female SEZ6L hets and is thus unlikely to account for the impaired motor coordination seen in female SEZ6L KOs. Like SEZ6L KOs, SEZ6 KO mice show deficits on the rotarod [19]. The more pronounced motor coordination impairments seen in (male) SEZ6 TKO mice tested on the rotarod [18], compared to single KOs, supports the idea of SEZ6 family members being functionally redundant as previously suggested [17]. Future experiments should determine whether motor deficits are similar in young and aged SEZ6L KOs as some motor deficits in SEZ6 TKO mice become more pronounced with age [18].

Overall, the motor phenotype of SEZ6L KO mice is similar to, but more subtle than, the phenotype of SEZ6 TKO mice. In contrast, results from the elevated open field are comparable in SEZ6L KO and SEZ6 TKO mice (Table 2), suggesting that SEZ6L deletion is contributing to the anxiety-like phenotype previously reported in SEZ6 TKO mice (which was also manifest in the zero maze, not examined in this study) [18]. Deletion of a single SEZ6L allele was sufficient to produce a clear change in locomotor behaviour on the elevated open field. Like SEZ6 TKO mice, SEZ6L KOs and hets showed a substantially lower level of movement and an increased latency to leave the start zone, indicating a reluctance to explore the stressful environment. Future studies should further investigate the role of SEZ6L in anxiety-related behaviour and identify whether SEZ6L KO mice have enhanced fear learning, as identified in SEZ6 TKOs [18]. Additionally, examination of a conditional SEZ6L KO, in which SEZ6L expression is deleted in adulthood, would reveal which cognitive and motor phenotypes are related to the absence of SEZ6L in the mature central nervous system.

SEZ6L is a single-span transmembrane protein with a large extracellular domain (ectodomain) containing three CUB (complement subcomponent C1r, C1s/sea urchin embryonic growth factor Uegf/bone morphogenetic protein 1) and five Sushi domains (also referred to as complement control protein or short consensus repeat (SCR)), followed by a transmembrane and a short intracellular domain. CUB and Sushi domains are features of proteins engaging in protein-protein interactions [40–43]. Moreover, SEZ6L is found at the surface of neurons [10, 44], suggesting that it may act as a surface receptor or ligand; however, the binding partners of SEZ6L are not yet known. SEZ6L has been shown to be a substrate of the transmembrane protease BACE1, which cleaves numerous membrane proteins in the nervous system [9, 15, 16, 22, 33, 45–49]. As a result of BACE1 cleavage, the large SEZ6L ectodomain is secreted into the conditioned medium of cultured cells or into body fluids, such as cerebrospinal fluid and plasma. This proteolytic process is referred to as ectodomain shedding and is a fundamental mechanism to control the function and abundance of surface membrane proteins [50]. Likewise, BACE1 cleavage controls the surface abundance of SEZ6L in primary neurons [10] but it remains to be seen whether this cleavage event also affects the function of SEZ6L, e.g. in motor coordination. Future experiments should examine the effect of BACE inhibitor treatment of SEZ6L KO mice or assess a BACE1-resistant SEZ6L knockin model. Nevertheless, BACE1-deficient mice also show deficits in motor coordination, linked to the reduced cleavage of another BACE1 substrate, the Ig-containing β1 neuregulin 1 (Nrg1). BACE1 cleavage of Nrg1 is required for motor coordination through the formation and maintenance of muscle spindles [11]. Potential motor deficits were also seen in Alzheimer patients and elderly individuals with high risk for Alzheimer’s disease that were treated in phase 3 studies with a BACE1 inhibitor [4, 5]. The treated individuals showed a small but significant increase in the number of falls, consistent with the motor deficits seen in BACE1-deficient mice. Whether the loss of BACE1-shed SEZ6L ectodomain also contributes to the motor deficits in BACE1-deficient mice, or in BACE1 inhibitor-treated individuals, remains to be determined.

Taken together, the results of our study establish a physiological function for SEZ6L in motor coordination and a pathological contribution of the lack of SEZ6L to anxiety-related behaviour, implicating aberrant SEZ6L function in movement disorders and neuropsychiatric diseases.

## Supplementary Information

Below is the link to the electronic supplementary material.

Supplementary file1 (PDF 1091 KB)

Supplementary file2 (XLSX 2841 KB)

## Data Availability

The datasets generated during and/or analysed during the current study are available from the corresponding author on reasonable request.
