# Supplementary material for: The β-Secretase Substrate Seizure 6–Like Protein (SEZ6L) Controls Motor Functions in Mice
Source: Mol Neurobiol. 2021 Dec 27;59(2):1183–98. doi: 10.1007/s12035-021-02660-y (PMC8857007; doi:10.1007/s12035-021-02660-y)
Supplement: Supplementary file 1 — Supplementary file1 (PDF 1091 KB) [file 12035_2021_2660_MOESM1_ESM.pdf]

Supplementary File

The β-secretase substrate Seizure 6-like protein (SEZ6L) controls motor functions in mice

Emma Ong-Pålsson*, Jasenka Rudan Njavro*, Yvette Wilson, Martina Pigoni, Andree Schmidt, Stephan A. Müller, Michael Meyer, Jana Hartmann, Marc Aurel Busche, Jenny M. Gunnersen, Kathryn M. Munro** and Stefan F. Lichtenthaler**

Supplementary Table 1 is provided as a separate Microsoft Excel file

Supplementary Table 2: DigiGait indices analysed with 2-way ANOVA with genotype and sex as factors to identify parameter changes that may be sex-specific. 15cm/s: WT n = 24 (9 male, 15 female), SEZ6L het n = 29 (14 male, 15 female) and SEZ6L KO n = 23 (15 male, 8 female). 25cm/s: WT n = 19 (6 male, 13 female), SEZ6L het n = 28 (13 male, 15 female) and SEZ6L KO n = 20 (13 male, 7 female). n.s. = not significant ($p>0.05$).

DigiGait indices	Treadmill speed	Examination of forelimb/paw or hindlimb/paw	Effect of genotype: p-value	Effect of sex: p-value	Interaction: p-value
Stance width (cm)	15 cm/s	Between forelimbs	n.s.	n.s.	n.s.
		Between hindlimbs	n.s.	n.s.	n.s.
	25 cm/s	Between forelimbs	n.s.	n.s.	n.s.
		Between hindlimbs	n.s.	n.s.	n.s.
Stride length (cm)	15 cm/s	Forelimb average	n.s.	n.s.	n.s.
		Hindlimb average	n.s. (0.0509)	n.s.	n.s.
	25 cm/s	Forelimb average	0.0133	n.s.	n.s.
		Hindlimb average	0.0061	n.s.	n.s.
Stride frequency (steps/s)	15 cm/s	Forelimb average	n.s.	n.s.	n.s.
		Hindlimb average	0.0200	n.s.	n.s.
	25 cm/s	Forelimb average	0.0197	n.s.	n.s.
		Hindlimb average	0.0059	n.s.	n.s.
Stride time (s)	15 cm/s	Forelimb average	n.s.	n.s.	n.s.
		Hindlimb average	0.0085	n.s.	n.s.
	25 cm/s	Forelimb average	0.0129	n.s.	n.s.
		Hindlimb average	0.0041	n.s.	n.s.
Stance duration (s)	15 cm/s	Forelimb average	0.0356	n.s.	n.s.
		Hindlimb average	0.0324	n.s.	n.s.
	25 cm/s	Forelimb average	0.0040	n.s.	n.s.
		Hindlimb average	0.0319	0.0198	n.s.
Swing duration (s)	15 cm/s	Forelimb average	n.s.	n.s.	n.s.
		Hindlimb average	0.0047	n.s.	n.s.
	25 cm/s	Forelimb average	n.s.	n.s.	n.s.
		Hindlimb average	0.0092	n.s.	n.s.
Propulsion phase (s)	15 cm/s	Forelimb average	n.s.	n.s.	n.s.
		Hindlimb average	n.s.	n.s.	n.s.
	25 cm/s	Forelimb average	n.s.	0.0054	n.s.
		Hindlimb average	n.s.	0.0017	n.s.
Brake duration (s)	15 cm/s	Forelimb average	0.0299	n.s.	n.s.
		Hindlimb average	0.0243	n.s.	n.s.
	25 cm/s	Forelimb average	n.s.	n.s.	n.s.
		Hindlimb average	n.s.	n.s.	n.s.

Supplementary Table 3: SEZ6L KO mice exhibit gait differences as assessed by treadmill walking at 15cm/s. p-values generated from 1-way ANOVA with male and female data pooled unless otherwise indicated. Tukey's multiple comparisons test significant differences between WT vs. SEZ6L KO indicted with an asterisk (*), SEZ6L het vs. SEZ6L KO indicated with a hash (#), and WT vs. SEZ6L het with a $. *p ≤ 0.05; **p ≤ 0.01; ***p ≤ 0.001 (or equivalent symbol). Data presented as mean ± SEM. WT n = 24 (9 male, 15 female), SEZ6L het n = 29 (14 male, 15 female) and SEZ6L KO n = 24 (15 male, 9 female). n.s. = not significant (p>0.05).

**DigiGait indices**	**Treadmill speed**	**Examination of forelimb/paw or hindlimb/paw**	**p-value**	**WT**	**SEZ6L het**	**SEZ6L KO**
Stance width (cm)	15 cm/s	Between forelimbs	n.s.	1.713 ± 0.045	1.734 ± 0.032	1.733 ± 0.069
		Between hindlimbs	n.s.	3.208 ± 0.061	3.255 ± 0.048	3.075 ± 0.056
Stride length (cm)	15 cm/s	Forelimb average	n.s.	5.13 ± 0.212	5.07 ± 0.072	5.43 ± 0.166
		Hindlimb average	0.0404	5.14 ± 0.177	5.08 ± 0.086	5.55 ± 0.153 #
Stride frequency (steps/s)	15 cm/s	Forelimb average	n.s.	3.11 ± 0.080	3.02 ± 0.044	2.87 ± 0.091
		Hindlimb average	0.0109	3.10 ± 0.073	3.04 ± 0.052	2.81 ± 0.080 * and #
Stride time (s)	15 cm/s	Forelimb average	0.0399	0.332 ± 0.008	0.339 ± 0.005	0.361 ± 0.011 *
		Hindlimb average	0.0044	0.333 ± 0.008	0.339 ± 0.006	0.370 ± 0.010 ** and #
Stance duration (s)	15 cm/s	Forelimb average	0.0188	0.221 ± 0.006	0.228 ± 0.003	0.244 ± 0.007 *
		Hindlimb average	0.0160	0.241 ± 0.006	0.246 ± 0.004	0.263 ± 0.006 *
Swing duration (s)	15 cm/s	Forelimb average	n.s.	0.111 ± 0.004	0.110 ± 0.003	0.118 ± 0.004
		Hindlimb average	0.0036	0.092 ± 0.003	0.093 ± 0.003	0.107 ± 0.004 ** and ##
Propulsion phase (s)	15 cm/s	Forelimb average	n.s.	0.151 ± 0.005	0.149 ± 0.003	0.157 ± 0.007
		Hindlimb average	n.s.	0.192 ± 0.006	0.182 ± 0.005	0.201 ± 0.008
Brake duration (s)	15 cm/s	Forelimb average	0.0385	0.071 ± 0.003	0.0795 ± 0.003	0.087 ± 0.006 *
		Hindlimb average	0.0250	0.049 ± 0.002	0.063 ± 0.004 $	0.061 ± 0.005

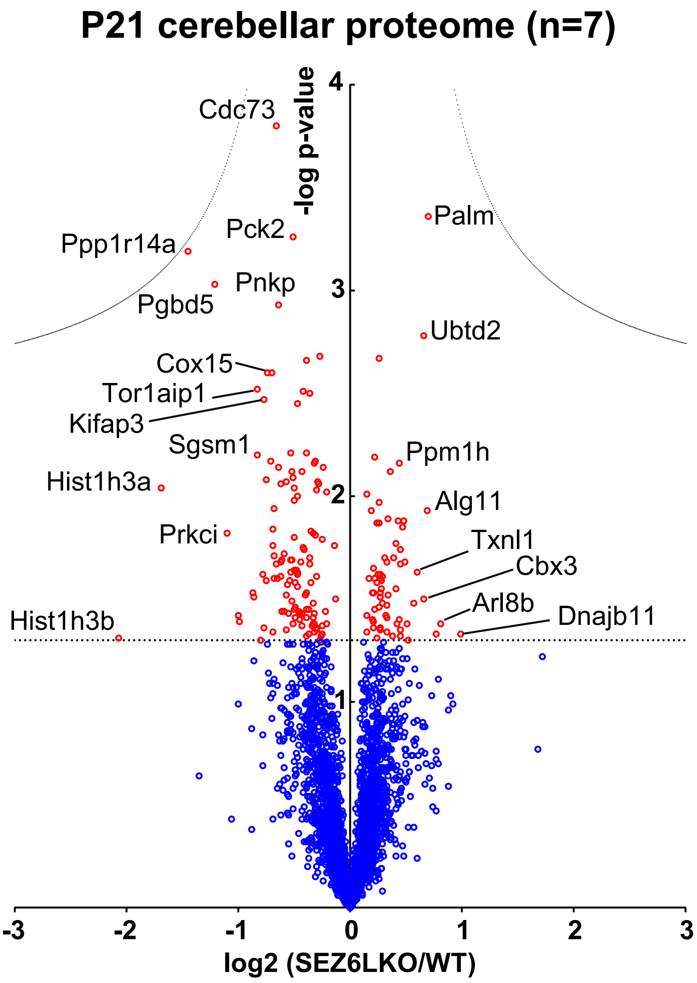

Supplementary Fig. 1

SEZ6L KO cerebellar proteome of the young mice. SEZ6L KO and WT cerebella at postnatal day 21 (P21) with seven biological replicates. The mean protein log2-transformed fold changes of each protein is plotted against the negative log10-transformed p-value. Proteins with a t-test p-value < 0.05 are shown as red circles, while the proteins with the p-value > 0.05 are shown as blue circles. Hyperbolic curve represents permutation-based FDR correction (FDR <0.05, s0= 0.1). The straight dotted line that crosses the y-axis at the 1.3 represents the p-value of 0.05.

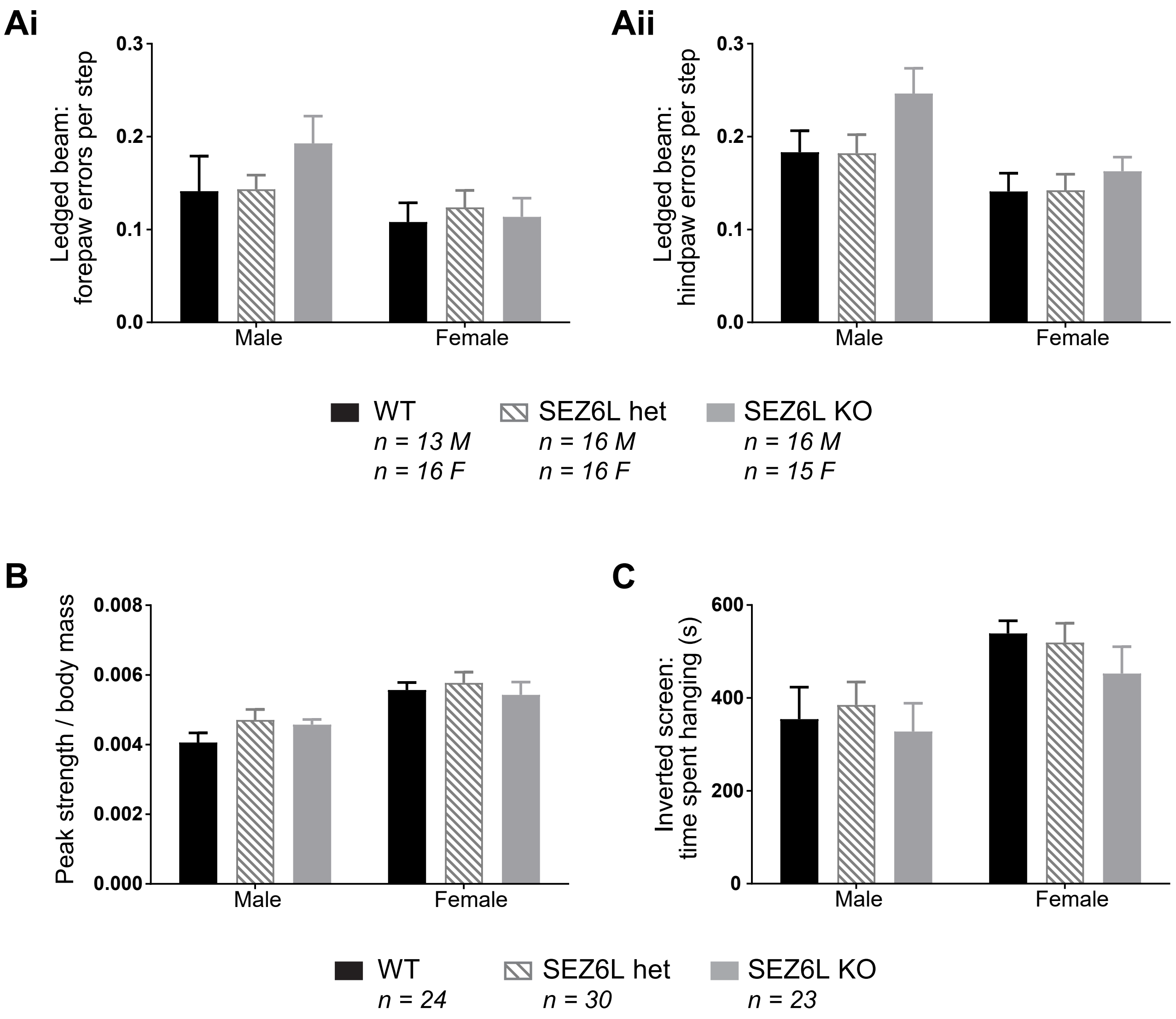

Supplementary Fig. 2

SEZ6L KO mice exhibit normal locomotor precision and muscular strength. WT, SEZ6L het and SEZ6L KO mice behave similarly in the ledged beam task (Ai, ii: 1-way ANOVA within sex), forelimb grip strength test (B: 1-way ANOVA within sex) and the four limb hanging inverted screen test (C: 1-way ANOVA and Kruskal-Wallis test within sex). Data shown as mean ± SEM. Mice tested on the ledged beam: WT n = 29 (13 male, 16 female), 32 SEZ6L hets (16 male, 16 female) and 31 SEZ6L KOs (16 male, 15 female). Mice tested on grip strength / inverted screen test: 24 WTs (9 male, 15 female), 30 SEZ6L hets (15 male, 15 female), 23 SEZ6L KOs (15 male, 8 female)
